# Cognitive Predictors of Precautionary Behavior During the COVID-19 Pandemic

**DOI:** 10.3389/fpsyg.2021.589800

**Published:** 2021-02-25

**Authors:** Volker Thoma, Leonardo Weiss-Cohen, Petra Filkuková, Peter Ayton

**Affiliations:** ^1^School of Psychology, University of East London, London, United Kingdom; ^2^Leeds University Business School, Leeds, United Kingdom; ^3^Department of High Performance Computing, Simula Research Laboratory, Oslo, Norway; ^4^Leeds University Business School, Leeds, United Kingdom

**Keywords:** COVID-19, cognitive reflection, cognitive failures, risk-taking, infection precaution, planned behavior

## Abstract

The attempts to mitigate the unprecedented health, economic, and social disruptions caused by the COVID-19 pandemic are largely dependent on establishing compliance to behavioral guidelines and rules that reduce the risk of infection. Here, by conducting an online survey that tested participants’ knowledge about the disease and measured demographic, attitudinal, and cognitive variables, we identify predictors of self-reported social distancing and hygiene behavior. To investigate the cognitive processes underlying health-prevention behavior in the pandemic, we co-opted the dual-process model of thinking to measure participants’ propensities for automatic and intuitive thinking vs. controlled and reflective thinking. Self-reports of 17 precautionary behaviors, including regular hand washing, social distancing, and wearing a face mask, served as a dependent measure. The results of hierarchical regressions showed that age, risk-taking propensity, and concern about the pandemic predicted adoption of precautionary behavior. Variance in cognitive processes also predicted precautionary behavior: participants with higher scores for controlled thinking (measured with the Cognitive Reflection Test) reported less adherence to specific guidelines, as did respondents with a poor understanding of the infection and transmission mechanism of the COVID-19 virus. The predictive power of this model was comparable to an approach (Theory of Planned Behavior) based on attitudes to health behavior. Given these results, we propose the inclusion of measures of cognitive reflection and mental model variables in predictive models of compliance, and future studies of precautionary behavior to establish how cognitive variables are linked with people’s information processing and social norms.

## Introduction

### Behavioral Measures to Control COVID-19

Countries world-wide are currently considering how to guide and change people’s behavior in order to maintain or ease COVID-related measures such as social distancing, increased hand washing, self-isolation, etc. These behavioral guidelines and the degree of their uptake are important to reduce the spread of the disease, prevent potentially very costly recurring waves of infections (Lauerman, 2020), and indeed mitigate likely future epidemics (or pandemics). Although compliance is not uniform, little is known about the psychosocial determinants of compliance with COVID guidelines (Bogg and Milad, 2020), and the current advice provided from scientists to policy-makers is based on general principles from pre-pandemic behavioral research (Bavel et al., 2020). Epidemiologists admit the lack of much-needed knowledge about the heterogeneity of behavioral responses (Weston et al., 2018). The famous Imperial College model (Ferguson et al., 2020), which altered the United Kingdom’s strategy, assumed 25% non-compliance on social distancing for people aged over 70, apparently without any specific empirical basis.

Nevertheless, studies conducted both before (Keizer et al., 2019) and during (Xie et al., 2020) the COVID pandemic have shown that various factors influence compliance with officially recommended health measures, which in turn should increase prevention success, including cognitive ability or disposition to pay attention, understand, memorize, or enact official guidelines. Thus far, however, no single study has investigated a comprehensive range of COVID-related precautionary behaviors and their dependence on multiple cognitive factors (see Xie et al. for the effect of working memory). It is possible that, when measured at a granular level (e.g., use of face masks and tracing apps), other cognitive factors may predict compliance with COVID-19-related precautionary measures. Consequently, the use of more fine-grained cognitive-behavioral predictions should enable better adherence estimates and allow adjustments of policies and guidelines (Anderson et al., 2020; Webster et al., 2020).

The current research investigates three specific cognitive variables – cognitive failures, cognitive reflection, and thinking disposition – and their potential role in precautionary measures during the COVID pandemic. These variables – together with knowledge about the new disease – were chosen because they relate to an important and often referred to theoretical framework, the so-called dual-processing theories (see, for reviews, Kahneman, 2011; Evans and Stanovich, 2013). The dual-processing theories propose that human judgment and decision behavior is driven by automatic and unconscious mental processes as well as by controlled and reflective thinking. While dual-processing theories seek to account for human thinking performance, another well-established theory offers potential for understanding and predicting COVID-19-related behaviors based on attitudinal differences is the Theory of Planned Behavior (TPB; Ajzen, 1991).

### Theory of Planned Behavior

The TPB has been applied to an extensive range of health-related behaviors (Armitage and Conner, 2001) and is the most influential social cognition model for predicting and explaining health behavior. TPB stipulates that a person’s behavioral, social, and control beliefs affect the intention for behavior change. For example, people who think that one cannot transmit the disease in the absence of observable symptoms will have behavioral beliefs (“Will this be effective?”), social beliefs (“Are others doing it?”), and control beliefs (“Am I able to do this?”), which make it less likely for them to adopt prevention measures.

TPB predicts an impressive 30–40% of the variance in health (prevention) behaviors (Armitage and Conner, 2001). TPB-related variables (attitudes and norms) can in principle be applied to COVID-19-related behavior (Sætrevik, 2020). Indeed, recent, but pre-COVID, research found that, in a Chinese sample, social norms, perceived behavioral control, and attitudes all predicted willingness to socially isolate in the face of a pandemic (Zhang et al., 2019).

### Dual-Process Theories of Thinking and Decision-Making

TPB notably assumes that attitudes and beliefs about actions are explicit, that is, they are given as a considered reflective account. However, within cognitive psychology, the dual-process theories of thinking and decision-making have become influential. They propose the workings in the mind consisting of both explicit reasoning and qualitatively different implicit judgment processes. The latter “Type 1” processing is thought to be fast, intuitive, and automatic, relying on heuristics (i.e., mental shortcuts) or “gut feelings” (Tversky and Kahneman, 1974); while the former explicit “Type 2” processing is considered to be slow, reflective, and effortful (Evans, 2003, 2010), encompassing logical and rational reasoning (Evans, 2008). Thus, unlike Type 2 processing, intuitive Type 1 processes are considered to be not under conscious cognitive control (Lowe Bryan and Harter, 1899; Shiffrin and Schneider, 1977; Evans, 2008), although the outputs from Type 1 thinking may or may not get overturned by conscious Type 2 processing (Kahneman and Frederick, 2002, 2007). While there are some critics of this notion (Gigerenzer and Regier, 1996; Osman, 2004; Keren and Schul, 2009; Kruglanski and Gigerenzer, 2011), the distinction between so-called Type 1 and Type 2 processes is supported by considerable empirical evidence (Evans, 1977; Evans et al., 1983; Klauer et al., 2000; Evans and Stanovich, 2013; Stanovich et al., 2019).

A more recent theory of dual-processing proposes a tripartite model that specifies two layers responsible for Type 2 processing: (1) the “algorithmic mind” and (2) the “reflective mind” (Stanovich, 2009). The performance of the algorithmic mind can be specified as the ability to override intuitive Type 1 responses and to respond with the correct analytical Type 2 responses (Toplak et al., 2011; see also Kahneman and Frederick, 2002, 2005). Its operations, therefore, should be related to attentional processes as well as mental simulation abilities (being able to separate and manipulate mental representational content, Stanovich, 2012). For example, the oft-used Cognitive Reflection Test (CRT, Frederick, 2005) presents a small series of brief math puzzles. Each of these CRT items evidently prompts an intuitively obvious – but incorrect – Type 1 answer; but, when applying reflective thinking, people are more likely to inhibit this first thought and produce the correct Type 2 answer by using basic algorithmic thinking. The CRT is thought to largely reflect the algorithmic layer processing in different ways (Stanovich, 2012): (1) it inhibits and overrides Type 1 (autonomous) processes and (2) it generates the correct answers by being able to symbolically manipulate representations (for which it needs attentional and working memory processing). For our dependent variable then, people may need to inhibit the automatic responses (i.e., it is easier not washing your hands so often, not wearing face masks, and not keeping extra distance). Furthermore, people may also need extra attentional and working memory processes (to remind oneself to wash one’s hands when coming into the house or when having touched surfaces, to memorize to stock and then find anti-bacterial gel, etc.). In fact, for some people (e.g., of older age), even the operation of different types of face masks may require instructions and significant efforts (Lee et al., 2020). Although the CRT is thought to be associated with a range of cognitive constructs (e.g., Toplak et al., 2011), including thinking dispositions and numeracy (e.g., Cokely and Kelley, 2009; Campitelli and Gerrans, 2014), recent evidence shows that working memory is the strongest single predictor of CRT performance (Stupple et al., 2017; Gray and Holyoak, 2020).

The reflective mind is the second layer within Type 2 processes and comprises higher-level cognitive styles, thinking dispositions, and metacognitive beliefs (Stanovich, 2011), which explain additional variance in thinking performance beyond the workings of the algorithmic mind (Stanovich and West, 2008; Stanovich, 2012). The reflective mind is responsible for the degree to which one thinks extensively about problems before responding, the amount of information one collects before making decisions, whether one integrates others’ points of view into one’s decisions or whether one adjusts beliefs according to the quality of the evidence (Baron, 2008). High actively open-minded thinking (AOT, Stanovich and West, 1998) scores have been shown to have a positive correlation with performance in the CRT (Baron et al., 2015) and belief bias syllogistic reasoning tasks (Macpherson and Stanovich, 2007). Thus, two people may have the same level of cognitive ability, but one may be more inclined than the other to engage their algorithmic mind because of their disposition to open-mindedly employ reflective thinking by taking in new information and be prepared to change their judgments based on it – a property of the reflective mind.

Based on the dual-process framework, and, in particular, Stanovich’s tripartite model, we hypothesized that people with higher cognitive reflection tendency (AOT) and ability (the algorithmic-level processing, measured with CRT) will engage more in thinking about, and therefore be more likely to employ, precautionary measures than people with lower cognitive reflection tendencies. Adopting new tasks, or performing them in a new context or with greater frequency (such as remembering to wash hands frequently and putting on face masks) should tax cognitive resources linked with the algorithmic mind, such as inhibition, attention, and working memory capacity (Stanovich, 2012). Indeed, cognitive reflection (measured with the CRT) has been shown to correlate positively with the ability to inhibit impulsive actions (Oechssler et al., 2009; Jimenez et al., 2018) and recently with causal learning task performance (Don et al., 2016).

In addition, people who perform better on the CRT have been found to be less susceptible to holding paranormal beliefs (Pennycook et al., 2012) and less prone to “unusual experiences” (generally linked to “jumping to conclusions”; Broyd et al., 2019). People with higher CRT scores also perform better at distinguishing fake from real news reports (Bronstein et al., 2019; Pennycook and Rand, 2019). Accordingly, since individual differences in willingness to engage effortful and reflective cognitive processes seem to be linked to propensity for irrational beliefs, then one can predict that people scoring low on the CRT will tend to be more likely to believe that the COVID-19 pandemic is a hoax, that risks are exaggerated, or that aspects of the guidelines are not to be believed. Consequently, they should be less likely to engage in precautionary behaviors, such as social distancing, wearing face masks, isolating, hand washing, etc. Indeed, Xie et al. (2020) have shown an effect of working memory on social distancing behavior – and since working memory performance is highly correlated with the CRT (e.g., Toplak et al., 2011; Gray and Holyoak, 2020), these data also strongly suggest a link between algorithmic thinking and precautionary behavior.

Furthermore, because adopting this range of behavior is effortful as one needs to change routines drastically, precautionary behavior should be observed more frequently when the underlying reasons are clear to the person (Bavel et al., 2020; Webster et al., 2020). More reflective people with a tendency to open-minded thinking (AOT) – a higher likelihood to inform themselves and adapt their judgments about the pandemic-related behaviors – are therefore predicted to take in new information about the pandemic and follow the official guidelines. There may, however, be a further reason why AOT would correlate with the uptake and compliance of precautionary behavior. This is the suggestion that people low on AOT scores tend to be politically more conservative, which is in some contexts (e.g., in the United States) associated with skepticism in government policies and official guidelines (Price et al., 2015; Baron, 2019). Allcott et al. (2020) employed United States geo-location data from smart phones and showed that republican-voting areas engage in less social distancing (controlling for other factors, including population density and local COVID cases). We therefore predict that people with more conservative leanings would score lower on the AOT and potentially also be less willing to adopt precautionary measures. To further disentangle cognitive inhibition performance (which the CRT measures) and thinking dispositions from general tendencies for impulsive behavior, we measure impulsivity and risk-taking tendencies separately.

### Attention and Cognitive Failures Questionnaire

Dual-process theories often refer to processes that are demanding of attentional and working memory resources when describing Type 2 thinking. However, attention and working memory are hardly ever tested directly in judgment and decision surveys.

People sometimes make mistakes even with rather mundane and familiar tasks, and common everyday failures can be measured by Broadbent’s cognitive failures questionnaire (CFQ; Broadbent et al., 1982, 1986a,b). The CFQ asks people to self-rate their propensity for slips of the mind that lead them to forget names, faces, or certain tasks. There is good evidence that the CFQ correlates with both self-reported and independently recorded errors and accidents (Wallace et al., 2003; Wallace and Chen, 2005; van Doorn et al., 2010; Day et al., 2012) and is associated with absentmindedness (Ishigami and Klein, 2009). Indeed, a recent systematic review of how CFQ self-report scores correlate with objective measures of executive function domains shows that CFQ is mainly associated with performance in selective attention (Carrigan and Barkus, 2016), rather than working memory or inhibition performance. Following the tripartite model of Stanovich (2011) and its explicit mention of attentional processes (Stanovich et al., 2019, p. 1118 and 1123), we included the CFQ as a measure of attentional capacity contributing to the algorithmic layer (Type 2) processing in addition to the measure of inhibition and simulation processes provided by the CRT.

That measuring differences in attention as an additional factor for predicting self-reported uptake of precautionary measures is reasonable is corroborated by evidence from field studies. A recent, but pre-COVID, review found that minimal hand-hygiene interventions at workplaces were effective in reducing the incidence of employee illness (Zivich et al., 2018). Almost all the interventions included in the review that effectively increased compliance involved drawing attention to hand washing and/or diminishing the load on people’s working memory.

### Mental Models

A further factor in predicting health behavior is the degree of knowledge and understanding of a disease. A recent review found that limited or insufficient health literacy was associated with reduced adoption of protective behaviors such as getting vaccinated (Castro-Sánchez et al., 2016). Sax and Clack (2015) also reviewed work showing that poor mental models affect uptake of hand hygiene in hospitals.

Mental models are representations of the world and its objects, the relationships between its various parts, and include perceptions about one’s own actions and their consequences. Mental models are distinct from mere knowledge or images, as they can contain abstract elements (Johnson-Laird and Byrne, 1991). Simply presenting people with scientific evidence does not mean that they fully understand – in a scientific sense – mechanisms of transmissions, prevention, and course of a (infectious) disease, because people form their own mental models about the biological and physical world influenced by their experiences and background knowledge. These conceptions often deviate substantially from scientific models (Legare and Gelman, 2008; Jee et al., 2015). For example, Sigelman (2012) found that when asked about the origins of the common cold, United States eighth graders (13–14 year olds) assigned cold weather explanations greater importance than germ-based explanations.

**Figure 1:**
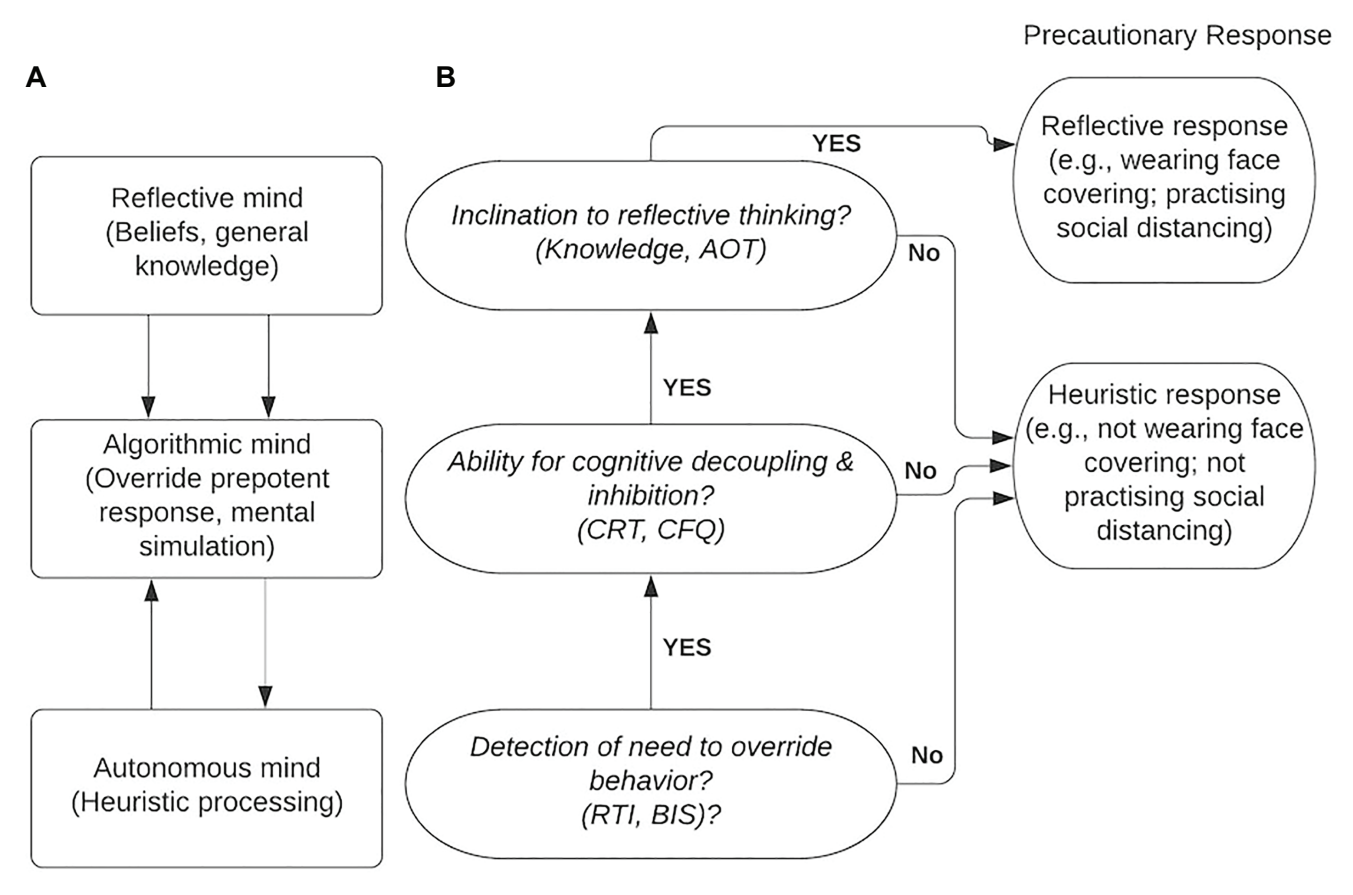
A tripartite model of thinking processes **(A)** adapted from Stanovich (2011) and its application in the current study **(B)**.

In our study questionnaires measure knowledge of COVID-19, asking which symptoms are related to COVID-19 (compared to common flu), questions probing the quality of the mental model of disease (transmission and immunity – again, compared to the common flu), prevention behavior (past and intended). Our predictions are that greater knowledge about COVID-19 symptoms and a better mental model about the disease transmission and prevention will correlate with better uptake of suggested precautionary measures. The logic of the study in terms of cognitive processes is summarized in Figure 1.

### The Current Study

Our survey measures cognitive variables related to dual-process frameworks, risk-taking, the knowledge or mental models people have developed about COVID-19, people’s understanding of the disease, and how these predict compliance with official prevention behaviors (including hand washing, wearing face masks, etc.). In addition, demographic (including political leanings) and experiential variables (such as media usage during the pandemic) were measured.

Following the dual-framework model, we hypothesize that cognitive reflection measures of the algorithmic level (CRT and CFQ) will predict uptake of the officially suggested COVID-19 prevention measures independently of demographic variables (age, sex, and concern about the pandemic) and impulsivity-related individual differences (risk-taking and behavioral inhibition tendencies). We also hypothesize that the amount of AOT, symptom knowledge and quality of the mental model of the disease will predict reported uptake of precautionary measures. To provide a baseline for assessing the explanatory power of the dual-process model, we will compare results with those from a simplified TPB model (which uses measures of different types of beliefs about the suggested behaviors) for predicting adherence to official behavioral guidelines.

## Method

### Procedure

The study used a cross-sectional quantitative design. Participants completed an online survey created using Qualtrics (2018). The data were collected on April 28, 2020. The order of questions is shown in Table 1. The data were analyzed with R 4.0.1 (R Core Team, 2020). The full questionnaire, datasets, R code and full results including additional analyses are openly accessible at the Open Science Foundation.^1^

**Table 1:** Descriptive results of the measures captured.

	Scale	Mean (SE) or *N*
**Demographics**		
Age	Continuous, in years	33.9 (0.73)
Gender = Female	Male and female	*N* = 206
Employed = Yes	Yes (Full time or part time)/No (incl. Student and retired)	*N* = 209
Politics	−3 = Left, 3 = Right-wing	−0.59 (0.07)
Main home = Yes	Yes/No	*N* = 289
Location = City	City/Country side	*N* = 213
**Covid-19 symptoms**		
S.Covid	Knowledge of symptoms of Covid-19, max. = 8 items	4.24 (0.09)
S.Flu	Knowledge of symptoms of Flu, max. = 8 items	3.53 (0.08)
S.Diff	Difference of Covid-19 minus Flu, max. = 8	0.71 (0.11)
**Covid-19 mental model**		
M.Covid	Mental model of Covid-18, max. = 8 items	7.45 (0.04)
M.Flu	Mental model of Flu, max. = 8 items	4.86 (0.07)
M.Diff	Difference of Covid-19 minus Flu, max. = 8	2.60 (0.09)
**Prevention methods**		
P.Not.Now (*α* = 0.87)	Preventive actions *not* being done, max. = 17	3.82 (0.21)
P.Effect	The actions are effective, −2 to +2	1.55 (0.04)
P.Follow	People are following these actions, −2 to +2	1.51 (0.04)
P.Easy	Ease of the preventive actions, −2 to +2	0.73 (0.06)
**Cognitive reflection**		
Cognitive Reflection Test (CRT; *α* = 0.74)	Correct answers, max. = 6	2.38 (0.11)
**Actively open-minded thinking (AOT)**		
AOT (*α* = 0.73)	Open-minded thinking: 1 = Disagree to 7 = Agree	5.15 (0.04)
**Impulsivity and risk-taking**		
Risk-Taking Index (RTI; *α* = 0.76)	Risk-taking: 1 = Never to 5 = Very often	1.81 (0.03)
Barratt Impulsiveness Scale (BIS; *α* = 0.78)	Impulsiveness: 1 = Never to 4 = Always	2.35 (0.03)
**Cognitive failures**		
Cognitive failures questionnaire (CFQ; *α* = 0.88)	Cognitive failures: 0 = Never to 6 = Very often	3.25 (0.06)
**COVID-related questions**		
News.Diff	After-before COVID-19 outbreak, in hours	0.57 (0.12)
Virus concern	1 = Not at all, 5 = A great deal	3.39 (0.06)
Isolation = Yes	Yes/No	*N* = 156
Positive diagnosis = Yes	Yes/No	*N* = 4

A simplified version of each scale used to capture the metrics is shown (see main text body for details). Results are shown either as the mean and standard error (in brackets) for continuous and scale measures or as the count (*N*) for binary measures. For the calculated scales P.Not.Now, CRT, AOT, RTI, BIS, and CFQ, Cronbach’s alphas are shown. Age, gender, and employment status were not asked directly but collected automatically from Prolific Academic’s database.

### Participants

We collected data from 300 participants surveyed online using Prolific Academic (female: *N* = 206; age: *M* = 33.89 years, SE = 0.72; see OSF for a *post-hoc* bootstrap power analysis). Only participants who were currently resident in the United Kingdom and had English as their first language were allowed to participate, using Prolific Academic’s pre-screening database. Participants were paid £2 and completed the questionnaire in an average time of 16.41 min (SE = 0.47). Two of the 300 respondents were omitted from the analyses due to missing age entries. Table 1 presents the means of all the measures captured.

### Measures

#### Demographics

Participants’ age, gender, and employment status were identified automatically from Prolific Academic’s database. Political leanings were assessed with one simple question: “Please choose the option that best represents your political views on a 7-point scale” from “Strongly left wing” to “Strongly right wing.”

#### COVID-19-Related Questions

We asked participants whether they were currently staying in their main home or somewhere else and whether this was in a city or the countryside. Two more brief questions established whether they were self-isolating during the last few weeks since the start of the pandemic and whether anybody in the household had tested positive for the virus. We also asked them questions about how many hours they spent on consuming news (traditional *via* papers, radio and TV, or online) before the pandemic and now during the pandemic to generate a score reflecting the self-reported change (News.Diff) in news consumption (after the pandemic minus before the pandemic). Finally, we asked “How concerned are you about your own personal safety and that of people close to you in terms of the virus?,” measuring respondents’ concern with a 5-point Likert scale (from “A great deal” to “not at all”).

#### Mental Models, Symptoms Knowledge, and Prevention Behavior

In order to evaluate participants’ knowledge of symptoms and their mental models related to COVID-19, we asked them two sets of questions related to symptoms and attributes of COVID-19. These items probed knowledge about the disease which was, in the period during and prior to the data collection, broadly disseminated by the national and international health organizations (Centers for Disease Control, 2020; National Health Service United Kingdom, 2020; Public Health England, 2020; Robert Koch Institute, 2020; World Health Organisation, 2020a) as well as official news media (Gallagher, 2020). As a part of the information about COVID-19 directed to the public, the differences between COVID-19 and flu have been highlighted (World Health Organisation, 2020b). Our participants were asked about symptoms and attributes (mental model) of flu in the same set of items which related to COVID-19.

#### Knowledge of Symptoms

Participants were provided a list of eight disease symptoms (fever, shortness of breath, dry cough, headaches, aches and pains, sore throat, fatigue, and runny or stuffy nose) and were asked to evaluate how frequently they occur in cases of COVID-19 and, separately, flu (answer options were “none,” “rare,” “sometimes,” and “common”). The correctness of their answers was evaluated according to the status of knowledge disseminated by media (e.g., CBS News, 2020; Woodward and Gal, 2020) and health authorities (Centers for Disease Control, 2020; National Health Service United Kingdom, 2020; Public Health England, 2020; Robert Koch Institute, 2020; World Health Organisation, 2020a) in March and April 2020. The symptoms score (S.Diff) was calculated as the difference between summed scores. Respondents were scored one point for each correct response, zero otherwise. The symptoms score (S.Diff) was calculated as the difference between summed scores for flu symptoms and COVID-19 symptoms.

#### Mental Models

In order to understand participants’ mental models of COVID-19 and flu, we listed eight statements pertaining to each disease, e.g., “there is a vaccine available”^2^ and “the symptom onset is gradual (rather than abrupt)” and asked participants to evaluate (yes/no) whether they apply to (a) COVID-19 and (b) flu. Again, the mental models score (M.Diff) was calculated as the difference between correct sum of scores for correct flu statements and the sum of COVID-19 knowledge. As with the symptoms above, our rationale was that the difference score would be more informative, assessing how much more (or less) people would know about COVID-19 compared to the well-known flu.

#### Prevention Behavior

To measure participants’ self-reported prevention behavior, we used a set of 17 items referring to COVID-19 prevention measures recommended by the authorities (e.g., “avoid touching surfaces in public” and “reduce using public transport”). For each of these items, participants reported dichotomously (yes/no) whether they (a) “currently do this or have recently (in the last two months)” and (b) “plan to do this from now on.”

Additionally, participants rated (from 1 = strongly disagree to 5 = strongly agree; a) perceived effectiveness of prevention behavior (“Do you agree that the actions mentioned above are effective?”), (b) its feasibility (“Do you agree with the following statement: ‘It will be easy to do these actions’”?), and (c) its application by significant others (“Do you agree with the statement: ‘In general, people important to you are following these actions’”?)

Two different presentation-orders of these three measures were randomly employed in the online questionnaire: (1) symptoms knowledge, (2) mental model of the disease, (3) prevention behavior and (1) prevention behavior, (2) symptoms knowledge, (3) mental model of the disease. There were no significant differences between these two conditions consequently in further analyses, the data were pooled.

#### Impulsivity and Risk-Taking

In order to control for the potential moderating effects of impulsivity, the Barratt Impulsiveness Scale (BIS) was administered (Patton et al., 1995). In the original version, participants respond to 30 items [on a four-point Likert scale from 1 (never/rarely) to 4 (almost always/always)]. We used an abbreviated scale of eight items based on the brief version of the scale by Steinberg et al. 2013. A sample item is “I don’t pay attention.”

The Risk Propensity Scale or Risk-Taking Index (RTI) was designed to assess risk preferences through a short self-report (Nicholson et al., 2005). Participants were asked to use five-point ratings (from 1 = never to 5 = very often) for six categories of risks: Recreational, Health, Career, Financial, Safety, and Social. These had to be rated twice: one for now and one for in the past, e.g., “We are interested in everyday risk-taking. Please could you tell us if any of the following have ever applied to you, now or in your adult past? – recreational risks (e.g., rock-climbing and scuba diving).”

#### Cognitive Reflection

The six CRT items were taken from two articles (Frederick, 2005; Toplak et al., 2014) excluding the “bat-and-ball” problem, due to its now high level of familiarity. A “decoy” item consisting of a simple mathematical problem (with no “lure” response) was shown as the first item (the “cargo ship problem”; Thomson and Oppenheimer, 2016), but did not contribute to CRT performance score. Respondents were asked to enter the correct number using their keyboard. Correct responses were scored with 1, while incorrect responses were given 0, and so the maximum total score was 6.

##### Sample Item

If it takes five machines 5 min to make five widgets, how long would it take 100 machines to make 100 widgets? ____ min (Correct answer: 5 min and intuitive answer: 100 min).

#### Cognitive Failures

The original CFQ consists of 25 items (Broadbent et al., 1982) arranged on a 5-point Likert scale (0 = never to 4 = always). Possible total scores range from 0 to 100 and Cronbach’s alpha for the scale has been found to be 0.90 and above, and it has been reported to have a test-retest reliability of 0.82 over a 2-month interval (Vom Hofe et al., 1998). We used a short form of the CFQ by Wassenaar et al. (2018), which retained 14 out of the original 25 items.

An example item is “Do you find you forget whether you’ve turned off a light or a fire or locked the door?”

#### Actively Open-Minded Thinking

AOT questionnaire (Baron, 1993; Haran et al., 2013) measures the willingness to consider new information and remain “open-minded.” Participants responded to items (e.g., Changing your mind is a sign of weakness) on a scale from 1 (completely disagree) to 7 (completely agree).

## Results

Two participants did not provide their age, so we omitted their data, leaving 298 respondents whose demographics and related background are summarized in Table 1.

The Cronbach’s alphas for the main psychological scales (AOT = 0.73, RTI = 0.76, BIS = 0.78, CFQ = 0.88, CRT = 0.74, and Prevention-Not-Now (P.Not.Now) = 0.87) ranged from acceptable to good (breakdown data for each question are available online at https://osf.io/8ahs5/).

We evaluated how well two different models predicted the extent of preventive behavior: a dual-process theory (DPT) model, and the TPB model. The dependent variable (DV) for each model was how many preventive measures against infection individuals reported as currently *not* doing, which was measured by the variable P.Not.Now. This is a count of “*not*” or “*negative*” answers and was coded as 1 for every “no” answer and 0 for every “yes” answer. The variable total score was calculated as the count of the 17 individual preventive methods and ranged from 0 (providing zero “*not*” answers, i.e., currently doing *all* the preventive methods) to 17 (providing 17 “*not*” answers, i.e., not currently doing *any* of the preventive methods).

Both models (dual-process, TPB) were evaluated using hierarchical regressions, with grouped blocks of independent variables being included sequentially. All the independent variables used in both models are shown in Table 2, and the correlation between them (we excluded potentially COVID-related variables that showed no significant association with the DV or the modeled predictors, such as News.Diff, living at home, political leanings, positive test of COVID, and employment situation – see OSF for the full correlation analysis).

**Table 2:** Pearson’s *r* correlation matrix for the variables used in the two analyses.

	Age	Gender	Concern	RTI	BIS	CFQ	CRT	AOT	S.Diff	M.Diff	P.Easy	P.Effect	P.Follow
Age	-												
Gender	−0.04	-											
Concern	0.11	−0.15^**^	-										
RTI	−0.13^*^	0.23^***^	−0.07	-									
BIS	−0.19^**^	−0.03	0.08	0.24^***^	-								
CFQ	−0.19^***^	−0.12^*^	0.09	0.17^**^	0.50^***^	-							
CRT	0.07	0.16^**^	−0.10	−0.01	−0.10	−0.08	-						
AOT	0.06	0.20^***^	−0.07	0.01	−0.17^**^	−0.07	0.26^***^	-					
S.Diff	0.11	−0.04	0.03	0.03	−0.06	0.01	0.01	0.04	-				
M.Diff	0.02	−0.14^*^	0.12^*^	−0.09	−0.01	−0.01	−0.10	0.00	−0.06	-			
P.Easy	0.15^**^	−0.05	0.06	−0.17^**^	−0.06	−0.09	−0.13^*^	−0.10	−0.03	0.06	-		
P.Effect	−0.03	−0.03	0.20^***^	−0.14^*^	−0.03	0.03	−0.01	0.06	−0.03	−0.04	0.29^***^	-	
P.Follow	−0.01	−0.09	0.14^*^	−0.04	−0.02	0.06	0.02	0.11	−0.05	0.01	0.20^***^	0.55^***^	-
Dual-process	X	X	X	X	X	X	X	X	X	X	-	-	-
TPB	X	X	X	X	X	-	-	-	-	-	X	X	X

The individual variables included in the dual-process and TPB models are identified below the table with an X. NB:

* *p* < 0.1;

** *p* < 0.05;

*** *p* < 0.01.

In both models, we included demographic predictors (including “concern for the virus”) in Block 1 and impulsivity and risk-taking indices (BIS and RTI, respectively) in Block 2. In the dual-process model cognitive variables related to algorithmic processing (CFQ and CRT) were tested in Block 3, and AOT and mental models [symptoms (S) and disease (M) – each as difference scores from flu, S.Diff and M.Diff, respectively], in Block 4. In the TPB regression, Block 3 contained the variables relating to beliefs about behavior. Table 2 (the last two rows) identifies the variables included in each model.

### Dual-Process Thinking

We started the analysis with a linear regression model (see OSF for additional results). However, P.Not.Now did not follow a normal distribution, and the fitted values from the linear model did not reflect the observed data (see OSF for histograms of observed and fitted data). In particular, the model did not predict any responses at zero (i.e., those with zero “*not*” answers, which equals full compliance with the list of preventive measures). This was in fact the most common answer.

Because of this excess (inflation) of answers at zero, and P.Not.Now being a count variable, we proceeded to fit the hierarchical model with a Zero-Inflated-Poisson (ZIP) model instead. While a standard Poisson model with the same average as our observed data would predict very few zero observations, the ZIP model attempts to better explain the excess observations at zero. It achieves this by using two separate processes to predict the final count of “*not*” answers: (1) a Poisson count model and (2) a binomial zero-inflated model. The main count model (1), which assumes a Poisson distribution, predicts the count of “*not*” answers (i.e., 0,1,2,3, etc.). This model mostly predicts a positive non-zero count (i.e., 1,2,3, etc.), with few zeroes; not enough to fit the observed data, which had an inflation of answers at zero. The excess of observations at zero is predicted by the zero-inflated model (2), which assumes a binomial distribution. This model predicts a binary outcome: it determines the probability of an individual answering with zero “*not*” responses or non-zero (i.e., one or more – the actual count is predicted by the Poisson count model). According to adjusted *R*^2^ and Akaike Information Criterion (AIC), the ZIP model fitted the data much better than the linear model (see OSF for a model fit analysis).

For the dual-process thinking analysis, the independent variables were added to the regression in sequential blocks, as shown in Table 3. The omnibus test of each additional block is also shown in Table 3, with every additional introduction of independent variables significant (*p* < 0.05).

**Table 3:** Independent variables included in each sequential stage of the dual-process thinking hierarchical regression analysis.

Block	Variables	Df	2 * ΔLL	*p*
0	Intercept			
1	Block 0 + Age + Gender + Concern	6	28.95	<0.001
2	Block 1 + RTI + BIS	4	26.52	<0.001
3	Block 2 + CFQ + CRT	4	18.57	<0.001
4	Block 3 + AOT + S.Diff + M.Diff	6	12.64	0.049

Each sequential block includes the variables from the previous blocks. The table shows the improvement in model fit from block to block, measured as twice the reduction in log-likelihood (how far away the fitted model is from the original data) in each additional stage.

The results of the models are shown in Table 4. The two processes can be interpreted separately. First, in the zero-inflated part of the model (which predicts zero or non-zero “not” answers), there was a significant effect of age, with older participants more likely to provide zero “not” answers (i.e., adopting all preventive methods), but no significant difference according to gender, in Block 1. More concerned participants were also more likely to provide zero “not” responses. In Block 4, there was a significant effect of the mental models of the virus (M.Diff). M.Diff measures how well participants understood the characteristics of the virus (compared to their understanding of the common flu virus). Participants who were more knowledgeable of the virus were more likely to provide zero “not” answers – i.e., adopt all preventive behaviors.

**Table 4:** Coefficients for the independent variables from each of the dual-process thinking hierarchical regressions.

	Block 1		Block 2		Block 3		Block 4	
**Count model (Poisson with log-link)**								
**Predictors**	**Log-mean (Std. Error)**	***p***	**Log-mean (Std. Error)**	***p***	**Log-mean (Std. Error)**	***p***	**Log-mean (Std. Error)**	***p***
(Intercept)	**1.91(0.13)**	**<0.001**	**1.20(1.21)**	**<0.001**	**1.00(0.22)**	**<0.001**	**1.11(0.34)**	**0.001**
Age	−0.01(0.00)	0.055	−0.00(0.00)	0.255	−0.00(0.00)	0.202	−0.00(0.00)	0.242
Gender (male)	0.03(0.06)	0.621	−0.03(0.07)	0.618	−0.07(0.07)	0.335	−0.06(0.07)	0.403
Concern	**−0.06(0.03)**	**0.045**	**−0.07(0.03)**	**0.018**	**−0.06(0.03)**	**0.033**	**−0.07(0.03)**	**0.028**
RTI			**0.23(0.06)**	**<0.001**	**0.22(0.06)**	**<0.001**	**0.23(0.06)**	**<0.001**
BIS			0.11(0.06)	0.061	0.12(0.07)	0.074	0.11(0.07)	0.105
CFQ					0.02(0.03)	0.626	0.02(0.03)	0.628
CRT					**0.06(0.02)**	**<0.001**	**0.06(0.02)**	**<0.001**
AOT							−0.02(0.04)	0.642
S.Diff							−0.02(0.02)	0.233
M.Diff							0.01(0.02)	0.685
**Zero-inflated model (Binomial with logit-link)**								
**Predictors**	**Log-OR (Std. Error)**	***p***	**Log-OR (Std. Error)**	***p***	**Log-OR (Std. Error)**	***p***	**Log-OR (Std. Error)**	***p***
(Intercept)	**−3.85(0.69)**	**<0.001**	**−2.80(1.11)**	**0.012**	−2.12(1.16)	0.068	−1.02(1.73)	0.555
Age	**0.03(0.01)**	**0.005**	**0.03(0.01)**	**0.012**	**0.03(0.01)**	**0.020**	**0.03(0.01)**	**0.018**
Gender (male)	−0.20(0.35)	0.561	−0.16(0.36)	0.651	−0.21(0.37)	0.569	−0.02(0.39)	0.966
Concern	**0.39(0.15)**	**0.008**	**0.39(0.15)**	**0.008**	**0.39(0.15)**	**0.008**	**0.35(0.15)**	**0.023**
RTI			−0.27(0.36)	0.445	−0.22(0.36)	0.544	−0.18(0.37)	0.621
BIS			−0.22(0.31)	0.480	0.06(0.35)	0.874	−0.02(0.35)	0.944
CFQ					−0.35(0.18)	0.053	−0.36(0.19)	0.061
CRT					−0.10(0.09)	0.251	−0.05(0.09)	0.582
AOT							−0.39(0.24)	0.110
S.Diff							0.05(0.08)	0.559
M.Diff							**0.33(0.12)**	**0.006**
N	298		298		298		298	
Adj. *R*^2^	0.263		0.395		0.447		0.465	

The dependent variable is always P.Not.Now. Coefficients are log-means for the Poisson model and log-mean of odds-ratios for the binomial model. Coefficients significantly different from 0 (with values of *p* below 0.05) are highlighted in bold. Two participants were excluded due to missing age data.

Second, in the count part of the model (which predicts the count of “not” answers), there was again a significant effect of concern in Block 1, with a negative coefficient; participants who were more concerned responded with fewer “not” answers (i.e., adopted more of the preventive behaviors). In Block 2, there was a significant effect of RTI, with more risk-taking participants who scored higher on RTI adopting fewer preventive behaviors, but no significant effect of BIS. In Block 3, there was a significant effect of CRT, with participants who scored higher on CRT adopting fewer preventive behaviors. There was no significant effect of CFQ. Overall, the observed *R*^2^ of the model was 0.46.

We also conducted a factor analysis, in order to better understand the relationship between the underlying individual responses which comprised P.Not.Now. We identified five factors based on shared correlations and common themes [(1) social distancing, (2) cleanliness, (3) mask usage, (4) sneezing protection, and (5) isolation]. We were particularly interested in the unusual correlation found with CRT. We found that the only factor which was positively correlated with CRT (i.e., the higher the CRT score, the higher the count of “not” answers) was factor 2 (cleanliness), with a correlation *r*(297) = 0.20, *p* < 0.001. This was confirmed by running the DPT models above on the biggest factors, factor 1 (social distancing – CRT is not a significant predictor, *p* = 0.116) and factor 2 (cleanliness – CRT is a significant predictor, *p* = 0.005; see OSF for more details on the factor analysis).

### Theory of Planned Behavior Analysis

We also evaluated a TPB model using a ZIP analysis, with the independent variables as shown in Table 5. All the individual steps of the analysis led to a significant improvement of model fit in comparison to the previous step.

**Table 5:** Independent variables included in each sequential stage of the TPB hierarchical regression analysis.

Block	Variables	Df	2 * ΔLL	*p*
0	Intercept			
1	Block 0 + Age + Gender + Concern	6	28.95	<0.001
2	Block 1 + RTI + BIS	4	26.52	<0.001
3	Block 2 + P.Easy + P.Effect + P.Follow	6	28.27	<0.001

Each sequential block includes the variables from the previous blocks. The table shows the improvement in model fit from block to block, measured as twice the reduction in log-likelihood (how far away the fitted model is from the original data) in each additional stage.

The results of the TPB analysis are shown in Table 6. Similarly to the previous model, in among the demographics included in Block 1 in the zero-inflated model, there was a significant effect of age, with older participants more likely to respond with zero “not” answers, but no significant difference according to gender. There was also a significant effect of concern, with more concerned participants also more likely to respond with zero “not” answers.

**Table 6:** Coefficients for the independent variables from each of the TPB hierarchical regressions.

	Block 1		Block 2		Block 3	
**Count model (Poisson)**						
**Predictors**	**Log-means (Std. Error)**	***p***	**Log-means (Std. Error)**	***p***	**Log-means (Std. Error)**	***p***
(Intercept)	**1.91(0.13)**	**<0.001**	**1.20(0.21)**	**<0.001**	**1.51(0.23)**	**<0.001**
Age	−0.01(0.00)	0.055	−0.00(0.00)	0.255	−0.00(0.00)	0.187
Gender (male)	0.03(0.06)	0.621	−0.03(0.07)	0.618	−0.03(0.07)	0.678
Concern	**−0.06(0.03)**	**0.045**	**−0.07(0.03)**	**0.018**	−0.05(0.03)	0.127
RTI			**0.23(0.06)**	**<0.001**	**0.18(0.06)**	**0.002**
BIS			0.11(0.06)	0.061	0.09(0.06)	0.149
P.Easy					**−0.11(0.03)**	**<0.001**
P.Effect					−0.02(0.05)	0.638
P.Follow					−0.07(0.05)	0.170
**Zero-inflated model (binomial)**						
**Predictors**	**Log-OR (Std. Error)**	***p***	**Log-OR (Std. Error)**	***p***	**Log-OR (Std. Error)**	***p***
(Intercept)	**−3.85(0.69)**	**<0.001**	**−2.80(1.11)**	**0.012**	**−3.31(1.23)**	**0.007**
Age	**0.03(0.01)**	**0.005**	**0.03(0.01)**	**0.012**	**0.03(0.01)**	**0.014**
Gender (male)	−0.20(0.35)	0.561	−0.16(0.36)	0.651	−0.21(0.37)	0.573
Concern	**0.39(0.15)**	**0.008**	**0.39(0.15)**	**0.008**	**0.37(0.15)**	**0.016**
RTI			−0.27(0.36)	0.445	−0.19(0.37)	0.610
BIS			−0.22(0.31)	0.480	−0.22(0.31)	0.480
P.Easy					0.03(0.16)	0.851
P.Effect					−0.13(0.28)	0.642
P.Follow					0.39(0.32)	0.226
N	298		298		298	
Adj. *R*^2^	0.263		0.395		0.491	

The dependent variable is always P.Not.Now. Results show the log-means for the Poisson model and log of odds-ratios for the Binomial model. Coefficients significantly different from 0 (with values of *p* below 0.05) are highlighted in bold. Two participants were excluded due to missing age data.

In the count model, in Block 2, there was also a significant effect of RTI, with more risk-taking participants who scored higher on RTI adopting fewer preventive behaviors, but no significant effect of BIS.

In Block 3, there was a significant effect of P.Easy, with participants adopting more preventive behaviors when they reported finding them easier. There was no significant of the P.Effect (how effective the behaviors were rated) or P.Follow (the extent to which their friends and relatives were also following the preventive measures). Overall, the observed *R*^2^ of the model was 0.49.

**Figure 2:**
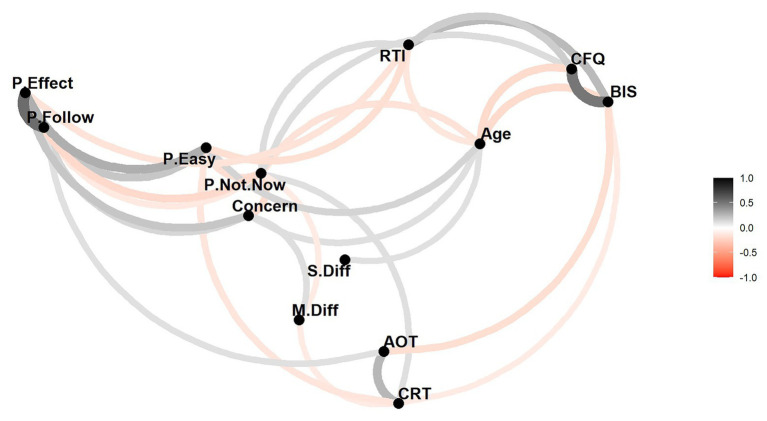
Visual representation of the correlations between the DV and all the IVs in both models (dual-process theory, DPT and Theory of Planned Behavior, TPB), similar to a network plot. Only correlations greater than 0.1 are plotted. Black lines indicate positive correlations and red lines indicate negative correlations. The darkness and thickness of the lines represent the strength of the correlation. The spatial location and proximity of the variables are determined by classical multidimensional scaling based on the absolute values of the correlations.

We then compared the two models according to AIC. The TPB model showed a slightly lower AIC (1550) than the DPM (1555), but the difference is small. Both models have a much better AIC than the linear regression model (see OSF for a model comparison analysis). Figure 2 illustrates the correlations (for coefficients *r* > 0.10) in both regression analyses (DPTM and TPB) between predictors and between the predictors and criterion (P.NotNow) in a network plot.

## Discussion

This online study is the first to our knowledge to test predictions from the DPT in the field of judgment and decision-making in relation to precautionary behavior in response to, and during, a pandemic. We found that cognitive factors, such as cognitive reflection and the quality of mental models (knowledge about the disease mechanism), predicted the amount of self-reported precautionary behaviors (including hand washing, wearing face masks, etc.) and hence compliance with official prevention guidelines.

The results from the first-order correlation analysis and subsequent hierarchical regression modeling are relatively clear: demographic factors previously associated with health behavior (Pack et al., 2001; Deeks et al., 2009), such as age (but not sex, see Branas-Garza et al., 2020 for a similar result regarding COVID-related donation behavior) as well as felt concern about the virus, explained a significant proportion of the variance on the DV, as did the RTI: older participants, respondents who were more concerned about the virus, and those self-reporting as less risk-taking in normal life, reported greater adherence to precautionary measures.

Interestingly, the cognitive reflection performance as measured by the CRT (even after accounting for thinking disposition, AOT) and measures of cognitive failures – which have not been used in the context of pandemic behavior, and hardly at all in the health behavior literature in general – correlated with preventive behavior: people reporting greater incidences of cognitive failures reported less behavioral adherence (although the individual contribution of CFQ observed in the first-order correlations is not significant anymore in the regression analysis). This would be predicted by standard cognitive theories, based on the notion that cognitive failures – as a proxy measure of attentional capacity – is linked to working memory (Heitz et al., 2005; Unsworth and Spillers, 2010; Oberauer, 2019) and hence to performance on tasks relying on such executive functions (McCabe et al., 2010; Xie et al., 2020).

Cognitive reflection performance as measured by the CRT uniquely predicted a portion of the variance in precautionary behavior. However, counter to our hypothesis, this correlation was negative – that is, people scoring lower on the CRT (and presumably leaning toward heuristics, fast judgments, and decisions) were more likely to engage in the recommended distance and hygiene measures to prevent the spread of COVID-19. In line with dual-process models as well as general conceptions about relevant health behavior tested in a pandemic (e.g., Bavel et al., 2020), we expected more reflective individuals to be more compliant, as, for them, the need for engaging in such demanding tasks – involving working memory and prospective memory (Xie et al., 2020) – should be easier to understand, plan, and adhere to. We discuss further possible explanations for this surprising finding below.

Finally, AOT and knowledge about the symptoms of the new disease did not predict reported behavior. AOT did, however, correlate positively with CRT – meaning that actively open-minded people are more prone to cognitive reflection, which is of course in line with the tripartite model (Stanovich, 2011).

### TPB and Cognition

The results of the current study show that TPB as a model of health-related behavior also predicted the uptake and maintenance of current precautionary behaviors at the first height of the COVID-19 pandemic. However, of the three behavioral attitudes only the variable of perceived behavioral control (the item measuring how easy it was to follow the behavioral advice) was a significant predictor (although the three attitudinal variables correlated with another). It is likely that, in this current pandemic, subjective norms were already at ceiling and that the vast majority was following the guidelines (early indications point to 83% compliance in the United Kingdom, Weinberg, 2020). Moreover, behavior compliance was relatively enforced (police checks on unnecessary travel) and alternative behavioral opportunities were already heavily curtailed (work places, entertainment venues, shops etc., closed).

Finally, it should be acknowledged that our TPB model was highly simplified, measuring behavioral attitudes with only three questions (perceived control, social norms, and effectiveness). Nevertheless, TPB predicted a substantial proportion of variance in precautionary behavior, explaining a similar amount of variance than the dual-process model.

### Explanations for CRT Correlation

Against our expectations, the correlation between CRT scores and avoidance of precautionary behavior was positive (i.e., the correlation between CRT and P.Not.Now was positive, with higher CRT scores correlated to more “not” answers to precautionary actions); more reflective people adopted fewer preventive behaviors. Our original expectation was based on the general notion that the tasks in the heuristics and biases literature are deliberately constructed to induce a heuristically triggered response, which needs to be overridden by a normative response generated by the analytic system. According to Stanovich’s concept of “cognitive decoupling,” the CRT measures the ability to inhibit automatic responses and simulate alternative responses (Stanovich, 2011). Our premise was that this ability would be needed if people were to adhere to precautionary measures, as they would need to override automatic responses, such as relying on their previous default behavior (Johnson and Goldstein, 2003) and in addition use mental simulation to employ the correct measures at the correct time, in the correct order. Similar reasoning has been invoked to explain why high CRT scorers are less likely to believe in conspiracy theories and fake news (Pennycook and Rand, 2019).

Concerning other correlations with the CRT, previous work has also reported effects of gender (Frederick, 2005; Campitelli and Gerrans, 2014; Thomson and Oppenheimer, 2016; Branas-Garza et al., 2020) using the classic three-item version, with male participants usually outperforming females. One reason given for this observation is that males have higher numeracy (Baron et al., 2015), though Campitelli and Gerrans cite both numeracy and rational thinking ability, whereas others think the difference could be due to higher anxiety or lower self-assessment on numerical aptitude (e.g., Zhang et al., 2016). Note that we omitted the notorious “bat-and-ball” question from the classic three-item test, which may have contributed significantly to the previously reported association with numeracy (Sinayev and Peters, 2015) and added four items from Toplak et al. (2014), which arguably are less reliant on numeracy. Less frequently reported are associations with age, with some authors finding no correlation (Campitelli and Gerrans, 2014; Thoma et al., 2015).

According to Baron (2017), the CRT is largely a measure of reflection/impulsivity: the willingness to take more time in order to be more accurate on judgment tasks, and CRT scores should therefore correlate with other normative responses. Clearly, this was not the case here for our type of responses, precautionary measures. Some commentators see the dual-system approach as only valid in well-structured environments such as psychological laboratory settings (Dane and Pratt, 2007; Hogarth, 2010; Magnusson et al., 2014). A similar argument is made by Risen (2016) who argues that Type 2 processing can be indeed differentiated as error detection and correction but adds the notion that error correction does not necessarily follow when an error is detected – and hence “acquiescence” is a possible System 2 response. This arguably explains why even “smart” people believe in magical thinking and superstition.

But although this approach may explain why we did not find a negative correlation between CRT and P.Not.Now, it does not explain why we still see a significant positive correlation between CRT and P.Not.Now. It is generally assumed that the CRT measures heuristic processing, and heuristics are thought to work through “attribute substitution”: when asked to answer a hard question (i.e., make numerical judgments) people substitute it with an easier one (e.g., “how easy does the answer come to mind?”; Kahneman and Frederick, 2002), which causes judgment biases. Able individuals’ Type 2 processing – measured with the CRT – will however, intervene and stop this substitution of a hard-to-evaluate characteristic for an easy one and usually improve judgment performance. However, according to West et al. (2012), when it comes to judgments about risks, Type 2 processing may do the opposite: “For example, people will substitute the less effortful attributes of vividness or salience for the more effortful retrieval of relevant facts. But when we are evaluating important risks—such as the risk of certain activities and environments for our children—we do not want to substitute vividness for careful thought about the situation. In such situations, we want to employ Type 2 override processing to block the attribute substitution.” (p. 508).

A different possible explanation could be that people with high CRT score thought more than others about the different guidelines and associated behavior, and in turn queried them critically to the point of higher non-adherence. For example, there is evidence that during an Ebola epidemic health professionals in quarantined villages were less likely to adhere to the quarantine than (presumably less knowledgeable) volunteers (see Webster et al., 2020). We originally hypothesized that the (perceived) effort of compliance with precautions would make less reflective people reluctant to adopt precautionary measures. However, conceivably, the effort of compliance may also spur the more reflective to think of reasons to override the prescribed behaviors; following precautionary guidelines, while effortful, may be *cognitively* simpler than generating reasons to dissent. If so then, accordingly, the non-compliant might conceivably be a mixture of two types: thoughtless recalcitrants (low on CRT) and thoughtful sceptics (high on CRT). The blend of each – and so the observed relationship between CRT and compliance – may depend on such things as the strength of social norms to comply (including how consistently experts endorse the measures) and how many other like-minded and/or critical people one is proximal to.

So could one have predicted these results if one assumes that irrational behavior (as measured by the CRT) depends on the perceived rationality or irrationality of the suggested measures by policy-makers and governments (for example, if people thought the measures were too drastic or even counterproductive then may be the positive CRT correlations express rational thinking)? Given that the data were collected at the height of the pandemic’s first wave (not only in the United Kingdom but also across Europe) and the measures (i.e., behavioral guidelines) we asked about apparently had a drastic effect in reducing infections, we think we rightly assumed that rational thinking and precautionary behavior were indeed linked at that time (in the first wave). Compliance in the population was very high then, and of course hygiene measures are widely accepted to be effective (although we now know that social distancing is even more important). Also, during the pandemic’s first wave many people have died, a strong argument for the rationality of these behavioral measures. Finally, the variable measuring concern did correlate positively with uptake of these measures.

Yet another possible explanation for the positive correlation between CRT and P.Not.Now is the negative association between CRT and prosocial acting. According to the recent study by Campos-Mercade et al. (2021), prosociality predicted health behaviors during the COVID-19 pandemic. According to Capraro et al. (2017), intuition is connected with concern for relative shares (which could be not only egalitarian but also spiteful), whereas deliberation is associated with individuals’ focus on social efficiency. In the context of economic games (e.g., the dictator game, the ultimatum game, and the prisoner’s dilemma), it was found that high cognitive reasoning and intelligence are negatively associated with cooperation and prosociality (Yamagishi et al., 2014) particularly in situations when the participants’ lack of cooperation did not have any negative consequences for them such as in one-shot games (Barreda-Tarrazona et al., 2017; Inaba et al., 2018). This association disappears in situations when cooperation has no or very low cost for the individual (Ponti and Rodriguez-Lara, 2015; Corgnet et al., 2016).

Based on these findings, prosociality was proposed to be connected with intuitive processes and the findings led to the social heuristics’ hypothesis, according to which intuition increases prosociality for people used to cooperative interactions (Rand et al., 2014; but see Chen et al., 2013; Verkoeijen and Bouwmeester, 2014). Clearly there is a need for further research to disentangle the significance of CRT scores from other psychological variables and contextual effects.

### Cognitive Failures Questionnaire

The CFQ correlated negatively with precautionary behavior, in the first-order correlations, although there was not a unique contribution of cognitive failures in the regression model. CFQ scores are related in the literature to variables, such as selective attention, multi-tasking, worry, stress, and boredom (Robertson et al., 1997; Wallace et al., 2003; Linden et al., 2005) – all factors that can be expected to play a major role in a lockdown situation in which many of the respondents will have found themselves in the United Kingdom. The main reason for including the CFQ was to enable us to disentangle cognitive reflection (CRT: cognitive inhibition and mental stimulation) from other cognitive processes (e.g., selective attention). Therefore, we cannot currently pinpoint a potential link between cognitive failures and precautionary behavior but given its association with a range of psychological factors, further research should be conducted to elucidate its role in preventative behavior.

### Mental Models

Knowledge of COVID-19 symptoms (S.Diff – comparing with knowledge of common flu symptoms) did not predict uptake of prevention behavior; however, the quality of the mental model around disease transmission and infection (M.Diff) did – similar to what was found, for example, for hospital staff (Sax and Clack, 2015). Regarding the lack of effects from symptoms knowledge, one possible reason could be a floor effect (median for P.Not.Now was 3, meaning that participants were doing 82% of all the possible actions) and that people were already well-informed at the height of the pandemic. Indeed, we did not find a correlation between P.Not.Now and additional (since the pandemic) news consumption *r*(298) = −0.02, *p* = 0.73. Future research will need to address the cause-and-effect relationship between cognitive reflection, mental models, and preventative behavior, but our results make it clear that the quality of information and their uptake by the population have a significant effect on compliance.

### Strengths and Limitations

Although based on theory, this study was necessarily exploratory to some degree, simply because of the novel nature of human actions it was investigating: the first global pandemic for 100 years. There are a number of variables that may have shed more light on our findings, e.g., perceived behavioral barriers (influences that discourage adoption of the behavior); also including an explicit measure self-efficacy (as often used within TPB) and measures of altruistic tendencies could help to find explanations for the patterns observed here. Nevertheless, the current research has some significance and originality, as it combines variables from two major theoretical strands of health-related research, the dual-process framework and TPB and demonstrates how these theoretical ideas could help to predict precautionary behaviors, and by extension, save human lives in future.

A further limitation is that we have not included further cognitive control variables – such as numeracy or math skills, which may explain part of the variance in CRT (e.g., Cokely and Kelley, 2009) – to better disentangle the analytic processes associated with predicting precautionary behavior. Furthermore, other variables could have made a contribution to the behavioral scores such as level of education. Another limitation is of course the time frame, as we could not trace changes in perceptions and actions over time during the COVID-19 crisis. Our survey captured the United Kingdom respondents at the height of the first lockdown (end of April 2020), only after which (from May to June 2020) there was an easing of both the pandemic and behavioral guidelines in the United Kingdom. It is possible that certain correlations between cognitive factors and precautionary behavior may be dependent on the length of time in which the measures have been already implemented. For example, it is possible that there would be a negative – instead of the observed positive – correlation between CRT and P.Not.Now in the early days of lockdown, when more reflective individuals may have assessed the situation as graver than the non-reflective.

### Conclusions

In a recent Nature Human Behavior perspective article (Bavel et al., 2020) by over forty behavioral scientists reviewing how insights from the social and behavioral sciences can be used to help align human behavior with the recommendations of epidemiologists and public health experts, the authors stressed the need for prosocial messages: e.g., “Leaders and the media might try to promote cooperative behavior by emphasizing that cooperating is the right thing to do and that other people are already cooperating.:…” Messages that (i) emphasize benefits to the recipient, (ii) focus on protecting others, (iii) align with the recipient’s moral values, (iv) appeal to social consensus or scientific norms, and/or (v) highlight the prospect of social group approval tend to be persuasive.” However, these authors did not mention cognitive reflection (or any other cognitive variables) as relevant factors.

In conclusion, our results demonstrate that individual differences in general cognitive abilities (cognitive reflection) and knowledge about the disease (mechanisms about transmission and infectiousness, but not knowledge about symptoms) are significant predictors for behavioral adherence to precautionary behavior in a pandemic, beyond known factors such as age or risk-taking. These variables appear to be as or even more predictive than differences in impulsivity, people’s political views, or where they live (town vs. country). This finding promises to close a gap in understanding compliance with precautionary behavior left by social norms approaches such as TPB.

People were more likely to adhere to official guidelines during the extraordinary COVID-19 pandemic when they were, in general, less reflective in their judgment and decision-making style, possibly due to them following heuristics or simple rules as this was an easier cause of action, they overly criticized the rationality of the guidelines or because they were following social norms. At the same time respondents were also more likely to follow these guidelines when they had a better understanding of the infection mechanism. Future research on cognitive factors in health-prevention behaviors should better establish how cognitive variables are linked with people’s information processing and social norms in order to improve predictions of precautionary behavior.

## Data Availability Statement

The datasets presented in this study can be found in online repositories. The names of the repository/repositories and accession number(s) can be found online at: https://osf.io/8ahs5/.

## Ethics Statement

The studies involving human participants were reviewed and approved by the City University of London. The patients/participants provided their written informed consent to participate in this study.

## Author Contributions

VT and PA conceived of the study, developed the theoretical background, planned and supervised the study, and wrote the article. VT, PA, and PF developed and designed the materials. LW-C ran the survey and analyzed the data and was the main contributor for the results section. PF contributed to literature search, theoretical discussions, write-up, and checking of the manuscript. All authors contributed to the article and approved the submitted version.

### Conflict of Interest

The authors declare that the research was conducted in the absence of any commercial or financial relationships that could be construed as a potential conflict of interest.

## Footnotes

^1^https://osf.io/8ahs5/

^2^At the time of the study there was no vaccine available for COVID-19.
